# Total Flavonoids from Snow Chrysanthemum Exert Synergistic Vascular and Neuroprotective Effects in Hypertensive Vascular Dementia Rats

**DOI:** 10.3390/ph19050700

**Published:** 2026-04-29

**Authors:** Xinyan Wu, Kangmeng Sun, Xinyu Wang, Mengying Hu, Xinyuan Sun, Baoping Jiang, Yuhua Sun, Chunnian He

**Affiliations:** 1 State Key Laboratory for Quality Ensurance and Sustainable Use of Dao-di Herbs, Institute of Medicinal Plant Development, Chinese Academy of Medical Sciences & Peking Union Medical College, Beijing 100193, China; 2 Key Laboratory of Bioactive Substances and Resources Utilization of Chinese Herbal Medicine, Ministry of Education, Beijing 100193, China; 3 Xinjiang Institute of Materia Medica, Urumqi 830002, China

**Keywords:** *Coreopsis tinctoria* Nutt., total flavonoids, vascular dementia, hypertension, vascular-neuro protective effect, transcriptomics

## Abstract

**Background/Objectives**: Snow Chrysanthemum (*Coreopsis tinctoria* Nutt.), a traditional medicinal and edible plant rich in flavonoids (TFSC) with antihypertensive and neuroprotective activities, has unclear effects and mechanisms on vascular dementia (VaD) comorbid with hypertension, a key risk factor accelerating VaD. This study aimed to investigate TFSC’s ameliorative effects on cognitive impairment in hypertensive VaD rats and elucidate its holistic therapeutic mechanisms. **Methods**: Spontaneously hypertensive rats (SHRs) with unilateral common carotid artery ligation were used to establish the hypertensive VaD model. TFSC was intragastrically administered for 11 weeks. Systolic blood pressure (BP) and cerebral blood flow (CBF) were monitored; cognitive function was assessed via open field, novel object recognition and Morris water maze tests. Histopathological changes were evaluated by H&E and Nissl staining, serum oxidative stress and inflammatory markers were measured, and hippocampal transcriptome sequencing plus RT-qPCR was performed to identify key pathways and genes. **Results**: The chemical profile of TFSC was characterized, showing a total flavonoid content of 84.96%; 49 compounds were identified, 39 of which were flavonoids. TFSC reduced BP, improved CBF, alleviated cognitive dysfunction and neuronal damage, enhanced antioxidant capacity (increased SOD, CAT, GSH; decreased ROS), and exerted anti-inflammatory effects (reduced TNF-α, IL-1β, IL-6, Ang-II). It modulated multiple pathways, with the PI3K-Akt and MAPK pathways enriched, and validated key differentially expressed genes. **Conclusions**: This study provides preliminary evidence for the holistic therapeutic potential of TFSC against hypertensive VaD. With integrated vascular regulatory and neuroprotective effects, TFSC serves as a promising candidate for VaD by targeting both vascular risk factors and neuropathological damage.

## 1. Introduction

Vascular dementia (VaD) is a neurodegenerative disease caused by vascular factors [1,2]. Hypertension constitutes not only a primary modifiable risk factor for the onset of VaD but also the most prevalent comorbidity in clinical practice. Epidemiological studies have documented hypertension in approximately 65.9% of patients at the time of VaD diagnosis, a finding corroborated by autopsy data demonstrating a hypertension prevalence of approximately 74% [3,4]. Among all modifiable contributors to VaD pathogenesis, elevated blood pressure (BP) ranks second only to aging, highlighting its central role in disease progression [5]. Given its reversible nature, hypertension represents a compelling therapeutic target for VaD prevention and management. Evidence indicates that antihypertensive agents, particularly calcium channel blockers and renin-angiotensin system inhibitors, may lower the incidence of dementia, including VaD [6]. However, monotherapy with these agents shows limited ability to halt or reverse established cognitive decline in VaD. Although combination regimens incorporating antihypertensive medications and cognitive enhancers have demonstrated clinical benefits, the resultant polypharmacy increases the risk of adverse drug reactions and compromises long-term patient treatment adherence [7]. In this context, medicinal plants with documented antihypertensive properties, such as *Eucommia ulmoides* [8], *Uncaria rhynchophylla* [9], and *Ginkgo biloba* [10], have garnered research interest. Their multi-target mechanisms and favorable safety profiles offer a promising complementary or alternative approach within integrative treatment strategies [11,12].

Snow Chrysanthemum (SC), derived from the dried capitula of *Coreopsis tinctoria* Nutt., is a traditional medicinal plant widely consumed as herbal tea in China, historically used to clear heat, detoxify, promote blood circulation, resolve stasis, invigorate the spleen and stomach, and prevent cardiovascular and cerebrovascular disorders [13,14]. Modern pharmacological research has validated its dual edible and medicinal properties, primarily attributable to its rich content of bioactive flavonoids, which demonstrate broad-spectrum activities, including antidiabetic, cardiovascular-protective, antioxidant, anti-inflammatory, neuroprotective, antimicrobial, and antitumor effects [15,16]. The total flavonoids from Snow Chrysanthemum (TFSC) have been shown to exert significant antihypertensive effects through multiple mechanisms: they induce vasodilation, thereby improving vascular function, and in hypertensive murine models, they effectively modulate BP, partly by targeting the renin-angiotensin system [17,18]. These findings position TFSC as a promising candidate for the prevention and treatment of hypertension and associated cardiovascular pathologies. Emerging evidence suggests that TFSC may enhance cognitive function. The ethyl acetate extract of SC, enriched in flavonoid compounds, has been demonstrated to ameliorate learning and memory deficits in aged mice, while its aqueous extract improves scopolamine- and sodium nitrite-induced memory impairments in murine models [19,20]. Collectively, flavonoids constitute the principal bioactive constituents of SC and have been preliminarily established to possess both antihypertensive and cognitive-enhancing properties. Its safety has been rigorously evaluated under the Administrative Measures for New Resource Foods and confirmed to meet national food safety standards, granting it legal recognition as a common food ingredient (https://slps.cfsa.net.cn/xwfb/gzcx/PassFileQuery.jsp, accessed on 12 October 2025). Compared with chemically synthesized drugs, SC exhibits superior safety and fewer adverse effects, making it particularly suitable for long-term administration in chronic diseases. However, whether TFSC can simultaneously elicit these dual pharmacological effects in patients with VaD and concomitant hypertension, thereby offering a synergistic therapeutic strategy, remains to be rigorously validated through targeted in vivo and clinical studies.

This study aimed to investigate the ameliorative effects and holistic mechanisms of the total flavonoids from TFSC on cognitive function in hypertensive VaD. Following the chemical characterization of TFSC, its effects on blood pressure, cerebral blood flow, behavioral performance, and hippocampal histopathology were evaluated in animal models. The antioxidant and anti-inflammatory activities, along with the associated signaling pathways, were systematically elucidated by measuring serum markers of oxidative stress and inflammation, and by performing hippocampal transcriptome sequencing coupled with RT-qPCR validation. This study provides experimental evidence supporting TFSC as a potential multi-target therapeutic agent for hypertensive VaD.

## 2. Results

### 2.1. Qualitative and Quantitative Analysis of TFSC

TFSC was obtained by heating reflux extraction followed by column chromatography, yielding a crude extract with a final extraction rate of 14.13%. The total flavonoid content in TFSC, as determined by the aluminum nitrate colorimetric method, was 84.96%, confirming flavonoids as the primary constituents. Quantitative analysis using UHPLC-DAD identified five major flavonoid components: marein (21.92%), isookanin-7-O-β-D-glucoside (13.04%), okanin (6.03%), quercetagetin-7-O-β-D-glucoside (3.64%), and isookanin (2.03%). Together, these compounds accounted for 46.66% of TFSC (Figure S1).

**Figure 1:**
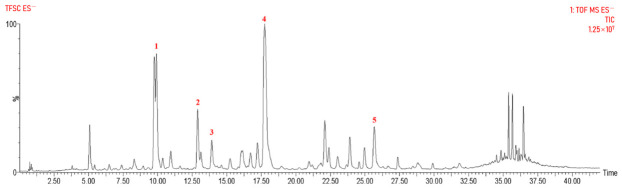
UHPLC-Q-TOF-MS/MS analysis of the chemical composition of TFSC. UHPLC-Q-TOF-MS/MS total ion chromatogram of TFSC in negative ion mode (ESI^−^). The identified peaks, confirmed by comparison with reference standards, correspond to isookanin-7-O-β-D-glucoside (**1**), quercetagetin-7-O-β-D-glucoside (**2**), isookanin (**3**), marein (**4**), and okanin (**5**).

To further characterize the chemical profile of TFSC, UPLC-Q-TOF-MS/MS analysis was performed. Based on the total ion chromatogram, 49 compounds were identified, with an additional 28 peaks tentatively annotated (Figure 1). These included 39 flavones, 5 phenylpropanoids, 2 phenolic acids, 2 polyacetylenes, and 1 terpenoid (Table S2). The results substantiate that flavonoids represent the predominant class of compounds in TFSC, and the identified major components provide a chemical basis for subsequent in vivo experiments.

### 2.2. Effect of TFSC on Blood Pressure and Cerebral Blood Flow in VaD Rats

**Figure 2:**
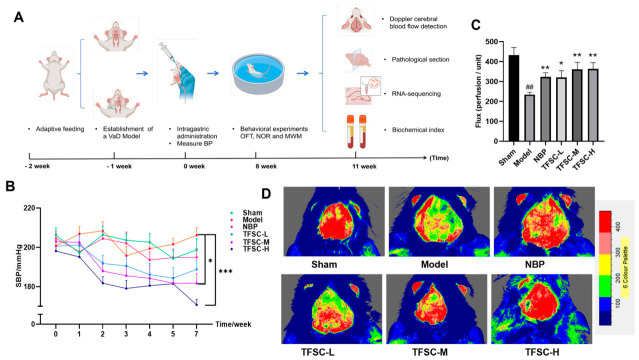
Effects of TFSC on BP and CBF in VaD rats. (**A**) Schematic diagram of the experimental designs of rats. (**B**) The trend of BP changes over the 0–7 weeks of drug administration. (**C**) Changes in CBF in each group after 11 weeks of treatment. (**D**) Representative laser Doppler flowmetry images of CBF in each group, measurement time 3 s. Data are presented as means ± SEM (*n* = 11–15). ## *p* < 0.01, compared with the Sham group; * *p* < 0.05, compared with the Model group; ** *p* < 0.01, compared with the Model group. *** *p* < 0.001 compared with the Model group.

As outlined in Figure 2A, VaD was induced in rats via unilateral carotid artery ligation after one week of acclimatization. Throughout the study, all animals remained healthy, and body weight increased steadily across groups (reaching a mean of 334.00 ± 15.12 g at Week 11; Figure S2) without significant intergroup differences (*p* > 0.05), indicating that neither the surgical procedure nor drug administration adversely affected general physiological condition.

Before treatment, baseline systolic BP was comparable among groups (*p* > 0.05), with values approximately 200 mmHg–significantly higher than the normotensive range reported for Wistar rats (108 ± 5 mmHg) [21]. After two weeks of treatment, BP remained elevated in the Sham, Model, and NBP groups, whereas a declining trend was observed in the TFSC-treated groups. By Week 2, the blood pressure (BP) of the TFSC-H group was significantly lower than that of the Model group (181.71 ± 3.36 mmHg vs. 208.40 ± 4.60 mmHg, *p* < 0.05; Figure 2B). In contrast, the positive control drug NBP did not elicit a comparable antihypertensive effect. The consistent downward trend indicates that TFSC effectively lowers BP, supporting its potential therapeutic utility in the management of VaD.

To further evaluate vascular responses, CBF was measured in the ischemic hemisphere. Compared with the Sham group (432.65 ± 38.29 PU), the Model group exhibited a significant decrease in CBF (234.39 ± 11.79 PU, *p* < 0.05), confirming that surgical ischemia induced sustained hypoperfusion. Both NBP and TFSC treatments significantly increased CBF relative to the Model group (TFSC-L: 320.627 ± 33.753 PU; TFSC-M: 360.96 ± 35.81 PU; TFSC-H: 364.11 ± 30.69 PU, *p* < 0.05; Figure 2C), indicating partial restoration of perfusion in affected brain regions. Representative laser Doppler perfusion images are shown in Figure 2D, where the color gradient (red to blue) reflects decreasing flow intensity. These results demonstrate that the VaD model successfully induced focal cerebral hypoperfusion and that TFSC treatment effectively attenuated this deficit, suggesting a protective action on cerebral circulation in VaD.

### 2.3. TFSC Ameliorates Cognitive Impairment in VaD Rats

**Figure 3:**
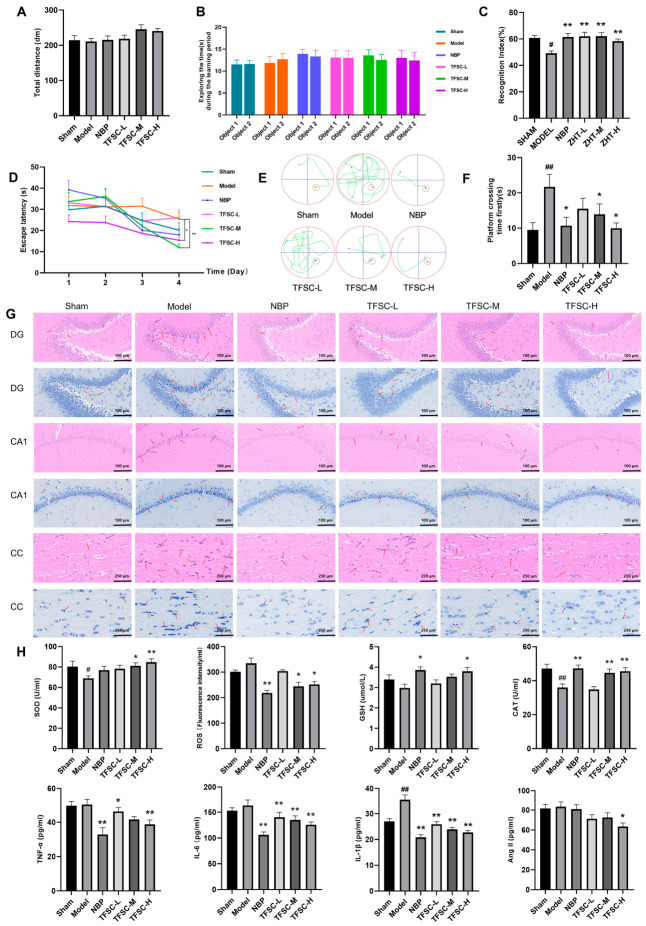
The effects of TFSC on cognitive impairment, brain tissue pathology, as well as oxidative stress and inflammatory indicators in VaD rats. (**A**) Average movement speed of rats in each group during the OFT experiment. (**B**) Number of explorations of two objects by rats in each group during the NOR familiarization phase. (**C**) Recognition index (RI) of rats in each group during the NOR testing phase. (**D**) Time taken by rats to reach the platform for the first time during the MWM training phase. (**E**) Representative trajectory maps of rats in each group on Day 4 of the MWM training phase. The red circle represents the original position of the platform, the red point represents the entry point of the rat, and the blue point represents the position where the rat reached the platform. (**F**) Time taken by rats to reach the platform for the first time during the MWM probe trial. (**G**) H&E staining (red) and Nissl staining (blue) in the hippocampal DG area, CA1 area, and CC area, with red arrows indicating abnormal nerve cells. (**H**) SOD, ROS, GSH, CAT, TNF-α, IL-6, IL-1β, and Ang II levels were measured in serum samples from each group. *n* = 11–15, data are presented as means ± SEM, # *p* < 0.05 compared with the Sham group, ## *p* < 0.01 compared with the Sham group, * *p* < 0.05 compared with the Model group, ** *p* < 0.01 compared with the Model group.

As shown in Figure 3A, no significant differences in total distance covered within 5 min were detected among groups in the OFT (*p* > 0.05), indicating that neither the surgical procedure nor drug treatments affected general locomotor activity.

During the training phase of the NOR test, no significant differences were observed in exploration times of Object 1 and Object 2 across all groups, indicating that the rats exhibited no inherent object preference and were suitable for subsequent testing (*p* > 0.05, Figure 3B). In the NOR testing phase, the recognition index was significantly decreased in the MODEL group (49.17 ± 1.74%) compared to the SHAM group (60.67 ± 1.96%, *p* < 0.05). TFSC treatment effectively reversed this deficit, as evidenced by the significantly increased recognition index in the TFSC-L (61.99 ± 3.07%), TFSC-M (62.07 ± 2.71%), and TFSC-H (58.27 ± 1.72%) groups compared to the MODEL group (*p* < 0.05; Figure 3C). The positive control drug NBP also showed a similar ameliorative effect (61.49 ± 2.77%). These results indicate that TFSC treatment enhanced the discrimination ability toward novel objects and improved non-spatial learning and memory capacities in VaD rats.

Spatial learning and memory were evaluated using the Morris Water Maze (MWM). On the first training day, escape latency did not differ significantly among groups (*p* > 0.05, Figure 3D). Over subsequent training trials, TFSC-treated rats showed progressively shorter latencies to locate the hidden platform compared with the Model group, indicating enhanced spatial learning (Figure 3D). On day 4, TFSC-treated rats showed a significantly shorter latency to locate the hidden platform compared to the model group (*p* < 0.05, Figure 3D), and representative swimming paths illustrate this improvement (Figure 3E). In the probe trial, the latency to first cross the former platform location was significantly prolonged in the Model group (21.72 ± 3.52 s) compared to the Sham group (9.53 ± 2.09 s, *p* < 0.05). Treatment with TFSC, particularly at the high dose (TFSC-H: 10.00 ± 1.50 s), as well as at medium (TFSC-M: 13.90 ± 2.98 s) and low doses (TFSC-L: 15.51 ± 2.95 s), significantly shortened this latency relative to the Model group (*p* < 0.05; Figure 3F). The positive control NBP (10.71 ± 2.39 s) also showed a similar protective effect.

Collectively, these behavioral results demonstrate that TFSC administration improves both non-spatial and spatial cognitive functions in rats with vascular dementia.

### 2.4. TFSC Ameliorated Histopathological Alterations in VaD Rats

Histopathological changes were assessed using H&E staining to evaluate nuclear morphology, cellular architecture, intercellular junctions, and membrane integrity, and Nissl staining to quantify neurons with intensely stained, abnormal Nissl bodies. In the Model group, marked histopathological alterations were observed in the hippocampal dentate gyrus (DG), cornu ammonis 1 (CA1), and corpus callosum (CC), including disorganized neuronal layers, aberrant cellular morphology, pyknotic nuclei, and an increased number of hyperchromatic Nissl-positive cells (Figure 3G). Treatment with TFSC significantly reduced the number of morphologically abnormal neurons and hyperchromatic Nissl-stained cells in both the hippocampal DG, CA1, and CC regions (Figure 3G). These results indicate that TFSC improves tissue pathology in VaD rats, suggesting a potential neuroprotective effect.

### 2.5. TFSC Ameliorates Oxidative Stress and Inflammatory Marker Levels in VaD Rats

The induction of VaD was associated with a compromised antioxidant defense system and inflammatory activation. Compared to the Sham group, rats in the Model group showed significantly reduced activities of the antioxidant enzymes SOD (Model: 68.97 ± 2.32 vs. Sham: 80.39 ± 5.35 U/mL, *p* < 0.05) and CAT (Model: 35.96 ± 2.14 vs. Sham: 47.11 ± 2.57 U/mL, *p* < 0.05), whereas levels of GSH and ROS remained unchanged (Figure 3H). Concurrently, the Model group exhibited a significant increase in the pro-inflammatory cytokine IL-1β (Model: 35.41 ± 1.89 vs. Sham: 27.03 ± 1.07 pg/mL, *p* < 0.05), while levels of TNF-α, IL-6, and Ang-II were not significantly altered. The lack of significant changes in GSH, ROS, TNF-α, IL-6, and Ang-II may be attributed to hypertension-induced elevations in baseline oxidative and inflammatory markers in Sham rats. TFSC treatment significantly counteracted these alterations. Specifically, TFSC enhanced antioxidant enzyme activities, increasing SOD (e.g., TFSC-H: 84.79 ± 3.21 U/mL), GSH, and CAT levels, and reduced ROS accumulation, exhibiting strong antioxidant capacity (*p* < 0.05, Figure 3H). In addition, TFSC markedly suppressed the secretion of TNF-α (e.g., TFSC-H: 38.76 ± 2.56 vs. Model: 50.34 ± 3.00 pg/mL), IL-6, IL-1β, and Ang-II, demonstrating significant anti-inflammatory effects (*p* < 0.05, Figure 3H). Collectively, these findings indicate that TFSC exerts potent antioxidant and anti-inflammatory activities in VaD rats.

### 2.6. TFSC Altered the Hippocampal Gene Expression Profile in VaD Rats

**Figure 4:**
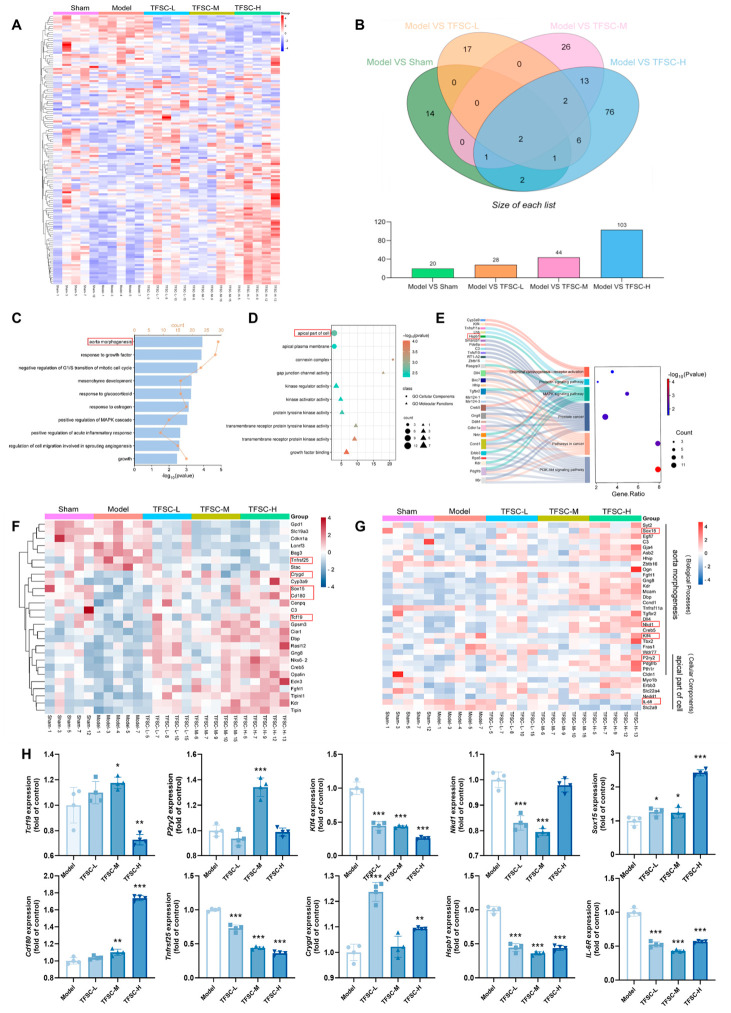
Differential gene expression and enrichment analysis in the hippocampus of VaD rats. (**A**) Heatmap of DEGs among the Sham, Model, and TFSC groups. (**B**) Venn diagram showing the number of DEGs for each group compared with the Model group. (**C**) GO enrichment results in the Biological Process category. (**D**) GO enrichment results in the Cellular Component and Molecular Function categories. (**E**) KEGG pathway enrichment results of DEGs. (**F**) Heatmap of common DEGs across the five groups. (**G**) Heatmap of DEGs involved in the “aorta morphogenesis” and “apical part of cell” GO enrichment analysis. The 10 genes highlighted with a red box were selected for RT-qPCR experimental validation. (**H**) RT-qPCR analysis of 10 key target genes, *n* = 4. Data are presented as mean ± SEM, * *p* < 0.05 compared with the Model group, ** *p* < 0.01 compared with the Model group, *** *p* < 0.001 compared with the Model group.

To investigate the molecular mechanisms underlying the therapeutic effects of TFSC in VaD, transcriptomic analysis was performed on hippocampal tissues. Comparative analysis between the TFSC-treated and Model groups identified 60 differentially expressed genes (DEGs), as visualized in a hierarchical clustering heatmap (Figure 4A). Venn diagram analysis revealed distinct expression profiles: 20 DEGs were identified between the Sham and Model groups, and 146 non-overlapping DEGs between the TFSC-treated and Model groups (Figure 4B). Among the TFSC-regulated DEGs, 100 genes were upregulated, and 46 were downregulated (Figure S3). These results demonstrate that TFSC significantly modulates the hippocampal transcriptome in VaD rats.

To elucidate the biological functions of the DEGs regulated by TFSC, GO and KEGG pathway enrichment analyses were conducted. GO analysis indicated that, within the Biological Process category, DEGs were primarily enriched in terms related to aorta morphogenesis, positive regulation of the MAPK cascade, and the acute inflammatory response (Figure 4C). For Cellular Component and Molecular Function, DEGs were mainly associated with the apical part of the cell, connexin complex, kinase regulator activity, and growth factor binding (Figure 4D). KEGG pathway analysis further identified six significantly enriched pathways, among which the PI3K-Akt and MAPK signaling pathways were predominant, with the former showing the highest enrichment score (Figure 4E). Heatmaps were generated to visualize the common DEGs across the five experimental groups (Figure 4F) and to highlight DEGs associated with the GO terms “aorta morphogenesis” and “apical part of cell” (Figure 4G). Collectively, these findings suggest that TFSC may exert its neuroprotective effects in VaD by modulating specific biological processes, cellular components, molecular functions, and key signaling pathways involved in vascular development, inflammation, and neuronal survival.

### 2.7. Validation of Key Gene Expression by RT-qPCR

To validate transcriptomic findings, quantitative RT-qPCR was performed on RNA extracted from the ischemic hippocampus of VaD rats. Ten key genes were selected for verification based on the following criteria: five represented common DEGs across all TFSC-treated groups (Figure 4F); five were associated with the GO terms “aorta morphogenesis” or “apical part of cell” (Figure 4G); and *Hspb1* was identified from KEGG pathway enrichment analysis (Figure 4E).

As shown in Figure 4H, TFSC treatment significantly downregulated the expression of *Klf4* (Model: 1.0 ± 0.05 vs. TFSC-H: 0.26 ± 0.01, *p* < 0.05), *Tnfrsf25*, *Hspb1*, and *IL-6R* compared to the Model group, indicating a broad inhibitory effect. For *Nkd1*, the downregulation was dose-selective, with significant suppression observed in the TFSC-L (0.83 ± 0.01, *p* < 0.05) and TFSC-M groups (0.79 ± 0.01, *p* < 0.05), but not in the TFSC-H group (*p* > 0.05). In contrast, TFSC consistently and significantly upregulated *Sox15* (TFSC-L: 1.26 ± 0.06; TFSC-M: 1.24 ± 0.08; TFSC-H: 2.42 ± 0.04, *p* < 0.05) and *Cd180* (TFSC-M: 1.10 ± 0.02; TFSC-H: 1.74 ± 0.01, *p* < 0.05) expression. Specifically, *Tcf19* showed increased expression in the TFSC-M group (1.17 ± 0.02, *p* < 0.05) but reduced expression in the TFSC-H group (0.73 ± 0.02, *p* < 0.05). *Crygd* was upregulated in the TFSC-L (1.24 ± 0.02, *p* < 0.05) and TFSC-H (1.09 ± 0.00, *p* < 0.05) groups, but not significantly in the TFSC-M group (*p* > 0.05). *P2ry2* expression was increased significantly only in the TFSC-M group (1.34 ± 0.04, *p* < 0.05). The results show that TFSC can regulate 10 differentially expressed genes in hypertensive VaD rats, and these genes may be potential targets for TFSC to exert its therapeutic effects.

## 3. Discussion

This study employed a composite model of hypertension combined with chronic cerebral hypoperfusion to investigate the pharmacological effects of TFSC in the treatment of VaD. The results demonstrated that TFSC administration in this model led to the following observations: a reduction in BP and an increase in cerebral blood flow in the ischemic hemisphere; an improvement in spatial and non-spatial learning and memory capacities, as assessed by the novel object recognition and Morris water maze tests; and an alleviation of histopathological damage in memory-related brain regions, including the hippocampal dentate gyrus and corpus callosum. Mechanistically, TFSC was found to enhance the activity of antioxidant enzymes (SOD, CAT, GSH), suppress the levels of pro-inflammatory cytokines (TNFα, IL1β, IL6) and Ang II, and alter hippocampal gene transcription. Taken together, these findings suggest that TFSC does not appear to target a single pathological mechanism in VaD. Instead, it may exert its therapeutic potential through simultaneous intervention in multiple interconnected pathological processes, including modulation of vascular risk factors and neuroprotective effects.

Integrating the findings of this study with existing literature, the ameliorative effect of TFSC on VaD may be achieved through a multi-level network. The core mechanism can be summarized as a dual intervention on “vascular system regulation” and the “nervous system protection”, with antioxidant and anti-inflammatory activities constituting the core pathophysiological bridge connecting these two major aspects [7,22]. Potential mechanisms of TFSC in improving VaD: a discussion based on a synergistic network of vascular regulation and neuroprotection.

### 3.1. Vascular System Regulation—Synergistic Effects on Improving Hemodynamics and Endothelial Function

The beneficial effect of TFSC on VaD may first stem from its multifaceted, interconnected regulation of the vascular system, centrally involving the synergistic effects of blood pressure modulation, improved cerebral blood flow, and protection of microvascular endothelial cells.

The antihypertensive effect demonstrated by TFSC is a crucial component of its intervention against vascular risk factors. This study found that TFSC administration lowered BP in VaD model animals while concurrently reducing Ang II levels. This effect may be partly attributed to modulation of the renin-angiotensin system (reduction in Ang II levels) [18]. Ang II is not only a potent vasoconstrictor but can also induce vascular oxidative stress and inflammation, impairing endothelial function [23]. Therefore, TFSC’s blood pressure-lowering effect may not only involve reducing peripheral vascular resistance but also mitigating the direct inflammatory vascular damage mediated by Ang II. Studies indicate a close pathophysiological link between BP abnormalities, changes in cerebral hemodynamics, oxidative stress, and neuroinflammation [24]. Chronic hypertension can lead to cerebrovascular remodeling and impaired cerebral autoregulation, consequently causing chronic cerebral hypoperfusion [25]. In turn, hypoperfusion can induce and exacerbate oxidative stress and neuroinflammatory responses, forming a mutually reinforcing vicious cycle that ultimately results in neuronal damage and cognitive decline [26]. The antihypertensive effect of TFSC may improve cerebral blood perfusion, reduce endothelial cell damage, and inhibit the cascade of oxidative stress and neuroinflammation exacerbated by hypertension. This mechanism helps maintain blood–brain barrier integrity, reduces the infiltration of neurotoxic substances, and thus provides a relatively stable internal environment for neural cells, indirectly exerting neuroprotective effects.

TFSC was able to increase CBF. This effect cannot be attributed solely to passive improvement following BP reduction but may also be closely related to its direct protective effect on the microvascular endothelium. Both chronic hypertension and cerebral hypoperfusion can lead to endothelial dysfunction [27]. In vitro experiments in this study confirmed that TFSC and specific flavonoid components can directly mitigate OGD-induced injury in cerebral microvascular endothelial cells. This suggests that TFSC may actively improve cerebral tissue blood perfusion by preserving endothelial integrity and ensuring normal vasomotor function and autoregulatory capacity. Improved cerebral perfusion restores energy and oxygen supply to neurons, helping to alleviate energy failure caused by ATP synthesis disorders [28], stabilize membrane potential, and thus reduce the massive release of excitatory amino acids (e.g., glutamate) and the excitotoxicity they mediate triggered by hypoxic depolarization at the source [29]. This optimized microenvironment resulting from vascular improvement provides a fundamental safeguard for neuronal survival and functional maintenance.

At the molecular mechanism level, transcriptomic and validation results from this study provide potential clues for the aforementioned vascular regulation. KEGG pathway enrichment analysis indicated that the PI3K/AKT signaling pathway was a core pathway highly enriched following TFSC intervention. The literature reports that this pathway can promote nitric oxide production by activating endothelial nitric oxide synthase in vascular endothelial cells, mediating vasodilation, and maintaining barrier function [30]. Combined with the observed increase in CBF and endothelial protective effects in this study, we speculate that TFSC may partly improve cerebrovascular function by activating this pathway. Further RT-qPCR validation identified some DEGs potentially related to vascular function, including *Klf4*, which is involved in endothelial homeostasis [31]. The P2Y2 receptor, encoded by the *P2ry2* gene, is a critical regulator of purinergic signaling. By mediating Ca^2+^ signaling in astrocytes, inflammatory activation, and the NF-κB pathway in vascular endothelial cells, it is extensively involved in the modulation of neurovascular coupling, BBB function, and the pathophysiology [32]. These changes suggest that TFSC may exert protective effects by regulating a gene network involved in vascular function and response. It must be noted that, because the transcriptomic data originated from hippocampal tissue containing mixed cell types, the changes in the aforementioned pathways and genes cannot be directly and specifically attributed to cerebrovascular cells. Their exact causal roles require future functional validation in specific cellular models.

### 3.2. Direct Neuroprotection—Structural-Functional Improvement and Potential Molecular Regulation

The most direct evidence for neuroprotection comes from behavioral improvements and histopathological observations in animal models. TFSC treatment significantly improved the performance of VaD rats in spatial and non-spatial memory tests, directly reflecting its ultimate effect on enhancing cognitive function. More importantly, histological analysis showed that TFSC alleviated neuronal abnormalities and structural damage in key memory-related brain regions, including the hippocampal dentate gyrus and the corpus callosum. These results directly demonstrate that TFSC intervention can mitigate neuropathological changes associated with learning and memory, constituting the core morphological and functional evidence for its neuroprotective action.

Transcriptomic analysis provides clues to the potential direct central mechanisms of TFSC. Gene expression profiling of hippocampal tissue revealed that TFSC modulated differentially expressed genes related to neuroprotection. For example, upregulation of the heat shock protein gene Hspb1 may help maintain neuronal protein homeostasis and resist the toxicity of misfolded proteins [33], while downregulation of Tnfrsf25 may attenuate pro-inflammatory and pro-apoptotic signals [34]. Concurrently, KEGG pathway enrichment analysis suggested the potential activation of signaling pathways, such as PI3K/AKT, which are closely related to cell survival, metabolism, and synaptic plasticity [35]. These molecular-level changes suggest that TFSC may directly exert neuroprotective effects by regulating specific gene and pathway networks within the central nervous system that are involved in cell defense, inflammation modulation, and survival. It must be emphasized that these findings are currently primarily correlative; their precise causal mechanisms require confirmation through subsequent gene-function studies.

### 3.3. Antioxidant and Anti-Inflammatory Effects—The Network Core Connecting Vascular and Neuroprotection

Based on the results of this study, the ameliorative effect of TFSC on VaD may be achieved through a network mechanism with antioxidant and anti-inflammatory activities at its core, connecting and traversing the two key pathological aspects of vascular system regulation and neuroprotection.

Firstly, the results of this study suggest that antioxidant and anti-inflammatory effects may underlie TFSC’s vascular protective actions. In the animal model, TFSC administration, while lowering BP and increasing CBF, was accompanied by elevated serum levels of antioxidant enzymes (SOD, GSH) and decreased levels of pro-inflammatory factors (TNF-α, IL-1β, IL-6). Oxidative stress and chronic inflammation can directly impair vascular endothelial function [36]. Therefore, the systemic antioxidant and anti-inflammatory effects of TFSC may synergistically promote its macroscopic hemodynamic improvements (e.g., lowering BP, increasing CBF) and help maintain blood–brain barrier integrity by mitigating oxidative and inflammatory assaults on the vascular endothelium. Secondly, these systemic effects may functionally link to central neuroprotection. The most direct neuroprotective evidence in this study is the improvement in cognitive behavior and the reduction of pathological damage in brain regions like the hippocampus in VaD model animals treated with TFSC. Although anti-inflammatory and antioxidant markers were measured peripherally, transcriptomic analysis in this study provides clues for its central action: TFSC intervention altered the expression of genes in hippocampal tissue related to stress response (e.g., Hspb1) [33], inflammation regulation (e.g., Tnfrsf25) [34], and cell survival pathways (e.g., PI3K/AKT) [35]. This suggests that TFSC may directly or indirectly enhance neuronal resistance to damage by modulating molecular networks involved in redox balance, inflammation, and cell survival within the central nervous system.

This study demonstrates that the therapeutic potential of TFSC should be evaluated within the broader context of current VaD management [37]. Current first-line pharmacological interventions, such as cholinesterase inhibitors and NMDA receptor antagonists, primarily target symptomatic relief and have limited ability to modify disease progression [38,39]. For the key modifiable risk factor of VaD—hypertension—clinical strategies often involve combination therapies of antihypertensive agents (e.g., calcium channel blockers) and cognitive enhancers (e.g., donepezil), yet polypharmacy may increase adverse reactions and compromise treatment adherence [7,40]. The findings of this study suggest an alternative approach. Our data indicate that TFSC achieves simultaneous multi-faceted intervention: it not only effectively lowers systolic blood pressure and improves cerebral perfusion (directly targeting the vascular etiology of VaD) but also directly alleviates neuronal damage, improves cognitive function, and suppresses associated neuroinflammation and oxidative stress, as confirmed by histopathological, behavioral, and serum biomarker analyses. Unlike single-target chemical drugs or complex combination regimens, TFSC, as a natural multi-component system, likely modulates multiple signaling pathways such as PI3K-Akt and MAPK through synergistic actions, thereby achieving multi-target intervention within a single agent. Under the experimental conditions, TFSC did not induce severe adverse effects, indicating a favorable safety profile. Therefore, TFSC represents a therapeutic strategy that integrates vascular protection with direct neuroprotection, offering a promising and potentially more manageable comprehensive treatment option for hypertension-associated VaD [40,41].

**Figure 5:**
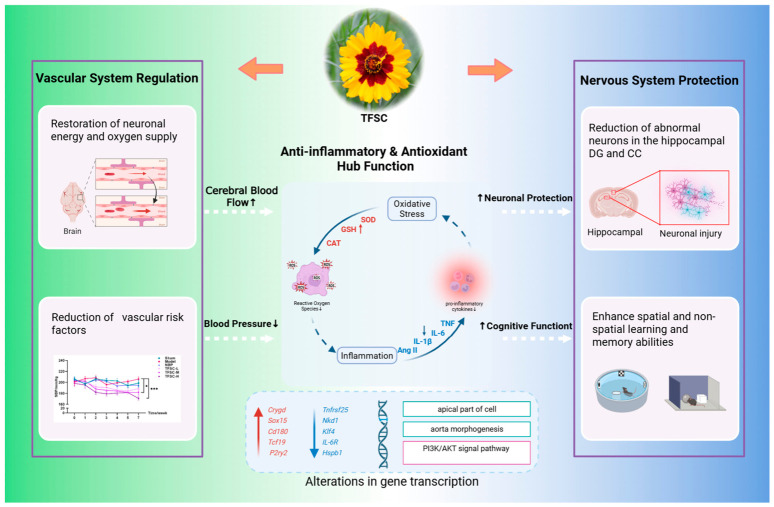
Schematic diagram of the mechanism of TFSC in the intervention of VaD. TFSC exerts dual vascular and neuroprotective effects and reduces pathological injury in VaD by regulating and interacting with anti-inflammatory and antioxidant pathways. Red arrows indicate upregulation, and blue arrows indicate downregulation; solid arrows indicate verified relationships, while dashed arrows denote hypothesized/unverified ones. * *p* < 0.05, *** *p* < 0.001. Part of this figure was generated using Biovisart (https://biovisart.com.cn, accessed on 1 November 2025).

Based on the research findings, the proposed mechanism of action of TFSC in the treatment of VaD is illustrated in Figure 5. The core mechanism involves a dual intervention targeting both the vascular system and the nervous system: at the vascular level, by modulating BP and improving CBF, it targets the vascular etiology of the disease; at the neural level, through its antioxidant and anti-inflammatory activities, combined with potential regulation of key hippocampal genes and signaling pathways (e.g., PI3K/AKT, Hspb1, Tnfrsf25), it directly mitigates neural damage and improves cognitive function. The antioxidant and anti-inflammatory effects constitute the core bridge connecting these two protective axes, achieving a synergistic effect by disrupting the vicious cycle of “vascular damage (BP) to cerebral hypoperfusion to oxidative stress/inflammation to neuronal injury”.

## 4. Materials and Methods

### 4.1. Medicinal and Reagents

N-Butylphthalide (NBP) soft capsules were obtained from CSPC NBP Pharmaceutical Co., Ltd. (Shijiazhuang, China). Detection kits for TNF-α, IL-6, IL-1β, Ang-II, SOD, GSH, CAT, and MDA were all purchased from Beijing Huaying Biotechnology Research Institute (Beijing, China). Fetal bovine serum, chromatographic grade acetonitrile, and chromatographic grade formic acid were acquired from Thermo Fisher Scientific Inc. (Shanghai, China). DMEM medium, 0.25% trypsin, PBS, and serum-free cell freezing medium were purchased from Beijing Solarbio Science & Technology Co., Ltd. (Beijing, China). The flavonoid standards including isookanin-7-O-β-D-glucoside, quercetagetin-7-O-β-D-glucoside, isookanin, marein, okanin, 7,8,3′,4′-tetrahydroxyflavanone, isocoreopsin, kaempferol-7-O-glucoside, butin, butein and quercetin-7-O-β-D-glucopyranoside were all obtained from Jiangsu Yongjian Pharmaceutical Technology Co., Ltd. (Taizhou, China).

### 4.2. Preparation of TFSC

SC was collected in August 2021 (at the flowering stage) from Keliyang Town, Pishan County, Hotan Prefecture, Xinjiang Uygur Autonomous Region, China, at an altitude above 3000 m. The plant material was identified as the dried capitulum of *Coreopsis tinctoria* Nutt. (*Asteraceae*) by Researcher Yuhua Sun from the Xinjiang Institute of Materia Medica. A voucher specimen has been deposited at the Center for Evolutionary and Phylogenetic Studies, Institute of Medicinal Plant Development, Chinese Academy of Medical Sciences. TFSC was prepared following a previously published study [42]: Dried plant material was extracted with 70% ethanol (1:10 *w*/*v*) via maceration at room temperature for 1.5 h, followed by three rounds of reflux extraction (1.5 h each). The combined filtrates were concentrated under reduced pressure at 50 °C. This extract (pH 5.0) was loaded onto a D101 macroporous adsorption resin column at 2.5 mL/min, and flavonoids were eluted with 5 bed volumes of 70% ethanol. The ethanol fraction was concentrated and lyophilized to obtain pure TFSC as a dry powder.

### 4.3. Determination of Total Flavonoid Content in TFSC

The total flavonoid content in TFSC was quantified using the aluminum nitrate-potassium acetate colorimetric method, with quercetin as the reference standard [43]. Briefly, 5 mL of the test solution was transferred into a 10 mL volumetric flask. Then, 0.3 mL of 10% Al(NO_3_)_3_ solution was added, mixed thoroughly, and allowed to stand for 6 min. Subsequently, 2.5 mL of 5% potassium acetate (KAc) solution was added, followed by an additional 6 min of incubation. Next, 2 mL of 4% NaOH solution was added, and the volume was adjusted to 10 mL with 70% methanol. After final mixing, equilibrate the solution for 15 min at room temperature. Absorbance was measured at 432 nm using a UV-Vis spectrophotometer, and the total flavonoid content was calculated from a quercetin calibration curve. A standard curve was established using quercitrin as the reference standard, plotting concentration (X) against absorbance (Y). The linear regression equation was Y = 0.0237X − 0.016 (R^2^ = 0.9998), with a linear range of 7.96–39.8 μg/mL.

### 4.4. Quantification of Five Major Flavonoids in TFSC

Representative flavonoids, including isookanin-7-O-β-D-glucoside (**1**), quercetagetin-7-O-β-D-glucoside (**2**), isookanin (**3**), marein (**4**), and okanin (**5**), were selected for quantitative analysis based on their high abundance in TFSC (Yongjian Biotechnology, Taizhou, China). Quantification was performed using an external standard method, with peak areas compared against certified reference standards [44,45]. Analysis was carried out on a Thermo Ultimate 3000 UHPLC system equipped with a DAD-3000RS detector (Thermo Fisher Scientific, Germering, Germany), using a Waters ACQUITY UPLC HSS T3 column (1.8 μm, 2.1 × 100 mm). The mobile phase consisted of solvent A (ultrapure water with 0.1% formic acid, *v*/*v*) and solvent B (acetonitrile with 0.1% formic acid, *v*/*v*). The elution procedure was programmed as follows: 0–1 min (5% B); 1–2 min (5–10% B); 2–6 min (10% B); 6–9 min (10–12% B); 9–15 min (12–15% B); 15–18 min (15% B); 18–25 min (15–20% B); 25–31 min (20–15% B); 31–36 min (22–40% B); 36–42 min (40% B). The flow rate was 0.3 mL/min, and the column temperature was maintained at 30 °C. The injection volume was 3 μL, and the detection wavelength was set to 378 nm.

### 4.5. UPLC-Q-TOF-MS/MS Analysis of TFSC

The qualitative profiling of TFSC was conducted using a Waters Acquity UPLC system coupled with a Xevo G2 Q/TOF mass spectrometer (Waters Corp., Milford, MA, USA), equipped with an electrospray ionization (ESI) source and operated in both positive and negative ion modes. UPLC conditions matched those used in Section 2.4. The optimized mass spectrometry parameters were as follows: desolvation gas flow rate, 800 L/h at 300 °C; cone gas flow rate, 50 L/h; and source temperature, 100 °C. The capillary voltage was 3.0 kV, and the sampling cone voltage was 30 eV. Data were acquired in MSE mode over a mass range of 150–1500 *m*/*z*, with a 1.0 s scan time. Collision energies were set to 6 eV (low) and 20–40 eV (high). Leucine enkephalin was used as the lock mass, providing reference ions at *m*/*z* 556.2771 ([M+H]^+^) and *m*/*z* 554.2615 ([M−H]^−^) for real-time mass correction. Data acquisition and processing were performed using MassLynx software (v4.1). Compound identification relied on: retention time alignment, accurate mass measurements, fragmentation patterns, comparison with authentic standards, and matching against an in-house SC compound database.

### 4.6. Study Design and Animal Experiments

The total experimental duration was 13 weeks, comprising 1 week of acclimatization, 1 week of postoperative recovery, and 11 weeks of drug intervention. The investigation commenced with the chemical characterization of TFSC. Subsequently, a hypertensive VaD model was established in SHRs by unilateral common carotid artery ligation. Drug administration began after a one-week postoperative recovery period (designated as Week 0). Body weight and systolic blood pressure were monitored weekly throughout the treatment period. Behavioral testing was initiated at Week 8, preceded by a 1-week acclimatization period in the behavioral testing suite. The tests were performed sequentially: OFT, NOR, and MWM. At Week 11, CBF was assessed using laser Doppler flowmetry, followed by terminal tissue collection. The therapeutic efficacy was evaluated by monitoring hemodynamic parameters (blood pressure and CBF), assessing cognitive and behavioral performance, and examining hippocampal histopathology via H&E and Nissl staining. To elucidate the underlying mechanisms, serum markers of oxidative stress and inflammation were quantified, and hippocampal tissues were subjected to transcriptome sequencing followed by RT-qPCR validation of key differentially expressed genes. During the experimental period, five rats died prior to terminal tissue collection (Model, Sham, NBP, TFSC-L, and TFSC-M groups; one each), primarily due to perioperative complications. A schematic overview of the experimental timeline is provided in Figure 2A.

Thirteen-week-old male SHRs with specific pathogen-free (SPF) status and an average body weight of 260 ± 20 g were obtained from Beijing Charles River Laboratory Animal Technology Co., Ltd. (Production License No.: SCXK (Beijing, China) 2021-0006). The animals were maintained in an SPF-grade facility at the Institute of Medicinal Plant Development, Chinese Academy of Medical Sciences. They had free access to food and water, under controlled environmental conditions: temperature 25 ± 2 °C, humidity 55 ± 10%, and a 12 h/12 h light-dark cycle. All experimental procedures were approved by the Ethics Committee of the Institute of Medicinal Plant Development, Chinese Academy of Medical Sciences (Approval No. SLXD-20230509021) and were conducted in compliance with relevant guidelines for the care and use of laboratory animals.

Following one week of acclimatization, rats were randomly assigned to six groups (*n* = 15 per group): (1) Sham group: received sham surgery and oral administration of 1.5% Tween-80 solution. (2) Model group: underwent VaD model surgery followed by oral 1.5% Tween-80 solution. (3) Positive control group: underwent VaD model surgery and received N-butylphthalide (NBP, CSPC-NBP Pharmaceutical Co., Ltd., Shijiazhuang, China) at 50 mg/kg/d. NBP was selected as the positive control because it is recommended for vascular cognitive impairment in the Chinese Guidelines for Diagnosis and Treatment of Vascular Cognitive Impairment (2019) [46] and shares multiple pharmacological activities (e.g., improving cerebral blood flow and reducing inflammation) [47] with TFSC, enabling a more relevant comparison. The rat’s equivalent dose (50 mg/kg/day) was derived from the standard human dose via body surface area normalization, prioritizing safety and experimental consistency [48,49]. (4–6) TFSC treatment groups: underwent VaD model surgery and received TFSC at low (50 mg/kg/d), medium (100 mg/kg/d), and high (200 mg/kg/d) doses, respectively. The theoretical effective dose of TFSC in rats (about 50 mg/kg/day) was calculated by extrapolating from typical human SC tea intake and adjusting via body surface area conversion factors. All groups except the Sham group underwent VaD model surgery.

### 4.7. Establishment of the VaD Rat Model

The VaD model was established by unilateral carotid artery ligation in spontaneously hypertensive rats (SHRs) to induce chronic cerebral hypoperfusion and subsequent cognitive impairment [50,51,52,53]. This model was selected to simulate the clinical condition of chronic cerebral hypoperfusion superimposed on a hypertensive background, a common subtype of VaD [54]. The SHR strain provides a stable hypertensive phenotype, while unilateral carotid artery ligation induces a sustained reduction in cerebral blood flow, replicating a key pathological feature of VaD progression. Rats were anesthetized with Zoletil 50 (3 mL/kg, Virbac Group, Carros, France) and placed in a supine position on a surgical platform. A midline incision was made in the neck to expose and carefully isolate the right common carotid artery, which was then ligated and transected, resulting in permanent occlusion of the artery. After suturing the incision, rats were monitored until fully conscious. In the sham-operated group, the right common carotid artery was exposed and isolated but not ligated, after which the incision was closed. Rats in the Model, positive control, and TFSC treatment groups underwent carotid artery ligation, whereas the Sham group received only the sham operation.

### 4.8. Blood Pressure Measurement

BP was measured non-invasively using the tail-cuff method with a BP-2010A system (Softron Beijing Incorporated, Beijing, China). Each rat was gently restrained in a warming chamber, and an inflatable cuff equipped with a pressure sensor was placed around the base of the tail. After the animal had acclimated and remained calm, the cuff was inflated to occlude the tail artery and then gradually deflated to record BP. The measurement was repeated three times, and the average value was calculated and recorded as the final BP reading.

### 4.9. Open Field Test

The OFT was conducted to assess spontaneous locomotor activity in rats [1]. The apparatus consisted of a black square arena (50 × 50 × 30 cm^3^). Each rat was gently placed in the center of the arena and allowed to acclimate for 1 min before a 5 min formal recording session began. Locomotor activity, including movement speed, was tracked and analyzed using the SuperMaze video tracking system (Shanghai Xinruan Information Technology Co., Ltd., Shanghai, China).

### 4.10. Novel Object Recognition Test

The NOR test was performed to evaluate non-spatial cognitive and memory function in rats, based on their innate exploratory preference for a novel object over a familiar one [55]. The test was conducted in a black open-field arena (50 × 50 × 30 cm^3^). Two identical cylindrical metal blocks (Object 1 and 2, each 5 cm in diameter) served as familiar objects, and a square prismatic metal block of similar size (Object 3) served as the novel object. The test consisted of three phases. Habituation: For three consecutive days, each rat was placed in the empty arena facing a wall, equidistant from the future object locations, and allowed to explore freely for 5 min without recording. Familiarization: Two identical cylindrical objects were placed symmetrically in the arena. The rat was introduced facing a wall, equidistant from object 1 and 2, and allowed to explore for 5 min. Test: 30 min after the familiarization phase, one familiar object was replaced with the novel object 3. The rat was returned to the arena, and its exploration of both objects was recorded for 5 min. All sessions were video-recorded, and exploration was analyzed using a video tracking system. Recognition index (RI) was calculated as follows: RI = (Time exploring the novel object)/(Time exploring the novel object + time exploring the familiar object).

### 4.11. Morris Water Maze Test

Spatial learning and memory were assessed using the MWM test, which measures a rat’s ability to locate a submerged hidden platform [55]. The apparatus consisted of a circular pool (1.2 m in diameter, 0.6 m in height) filled with water maintained at 25 ± 1 °C and rendered opaque with black dye. A cylindrical platform (10 cm in diameter) was submerged approximately 1 cm below the water surface in the center of one quadrant (designated the target quadrant). The pool was conceptually divided into four equal quadrants (I–IV). Acquisition Phase: Over four consecutive days, rats underwent two training sessions per day from different starting quadrants (excluding the target quadrant). In each trial, a rat was gently placed into the water facing the wall and given 60 s to locate the hidden platform. If the rat found and remained on the platform for 3 s, the escape latency was recorded. If it failed to find the platform within 60 s, it was gently guided to the platform and allowed to remain there for 10 s; in this case, the escape latency was recorded as 60 s. Probe Trial: On the fifth day, a probe trial was conducted to evaluate memory retention. The platform was removed, and each rat was released from the quadrant opposite the original platform location and allowed to swim freely for 60 s. The time to first cross the former platform location was recorded and analyzed.

### 4.12. Cerebral Blood Flow Measurement

Following anesthesia, rats were placed in a prone position. The scalp hair was shaved, and the skin was disinfected. A midline incision (approximately 1.5–2.0 cm) was made to expose the skull surface. The head was then positioned under the camera of a laser Doppler perfusion imager (T402-pp, Transonic, Ithaca, NY, USA). Cerebral blood flow (CBF) was monitored continuously for 30 s to achieve a stable signal. A representative image was then acquired over a 3 s acquisition period to minimize motion artifacts induced by respiration or other movements. CBF signals were acquired and analyzed using MoorFLPI Full-Field Laser Perfusion Imaging Review software (v4.0, Moor Instruments, Axminster, UK). After imaging, while the animals remained under anesthesia, tissue samples were collected for subsequent analyses.

### 4.13. Histological Staining (Hematoxylin & Eosin and Nissl Staining)

Following transcardial perfusion via the abdominal aorta, brain tissues were harvested and fixed in 4% paraformaldehyde. The tissues were subsequently dehydrated through a graded ethanol series, embedded in paraffin, and sectioned coronally at 5 μm. For hematoxylin and eosin (H&E) staining, sections were stained with hematoxylin, differentiated, blued, dehydrated through 85% and 95% ethanol, and counterstained with eosin. For Nissl staining, sections were immersed in cresyl violet solution, rinsed gently with distilled water, differentiated in 0.1% glacial acetic acid, dehydrated, cleared, and coverslipped. Histopathological evaluation of stained brain sections was performed using ImageJ (version 1.52).

### 4.14. ELISA Analysis

The pathogenesis of VaD is closely linked to neuroinflammation and oxidative stress, both of which significantly contribute to disease progression [56]. To evaluate these pathological factors, serum levels of inflammatory cytokines and oxidative stress markers were quantified using enzyme-linked immunosorbent assay (ELISA) kits from Beijing Huaying Biotechnology Research Institute (Beijing, China) according to the manufacturer’s instructions. Specifically, the concentrations of tumor necrosis factor-α (TNF-α), interleukin-6 (IL-6), interleukin-1β (IL-1β), angiotensin II (Ang-II), superoxide dismutase (SOD), glutathione (GSH), catalase (CAT), reactive oxygen species (ROS), and malondialdehyde (MDA) were measured. The absorbance of each well was measured at 450 nm using a DR-200BS microplate reader (Huaside Lang, Wuxi, China).

### 4.15. Transcriptome Analysis

For transcriptome sequencing, hippocampal tissues were randomly collected from five rats per group. Total RNA was extracted using a Trizol reagent kit (Thermo Fisher Scientific, Waltham, MA, USA). To construct the RNA library, mRNA was purified from total RNA by poly(A) selection, fragmented into 300–350 bp segments, and reverse transcribed into first-strand cDNA using dNTPs, followed by second-strand synthesis. PCR amplified the resulting double-stranded cDNA, and the products were purified to generate the final sequencing library. Paired-end sequencing (150 bp) was performed on an Illumina NovaSeq 6000 platform. DEGs were identified with the thresholds: |fold change| > 1.5, *p* < 0.05, and total expression count > 25. Functional enrichment analysis of DEGs were conducted using the Metascape database (https://metascape.org) with the following parameters: *p* < 0.05, minimum overlap = 3, and minimum enrichment = 1.5, to obtain Gene Ontology (GO) terms and Kyoto Encyclopedia of Genes and Genomes (KEGG) pathways.

### 4.16. Real-Time Quantitative PCR

Gene expression in the ischemic hippocampus was analyzed by reverse transcription followed by real-time quantitative PCR (RT-qPCR). Total RNA was extracted from the right hippocampus of each rat using the TRIzol-chloroform method with isopropanol precipitation (Takara, Osaka, Japan). cDNA was then synthesized from 1 μg of total RNA using a commercial reverse transcription kit (TransGen Biotech, Beijing, China). RT-qPCR was performed in a 20 μL reaction volume under the following thermal cycling conditions: initial denaturation at 94 °C for 30 s, followed by 40 cycles of 94 °C for 5 s, 57 °C for 15 s, and 72 °C for 10 s. Gene expression levels were normalized to a housekeeping gene and quantified using the 2^−ΔΔCt^ method. All primer sequences were designed based on target gene sequences retrieved from the NCBI database and are listed in Table S1.

### 4.17. Statistical Analysis

All data were analyzed using IBM SPSS Statistics software (version 22.0) and are expressed as mean ± standard error of the mean (SEM). The normality of data distribution was assessed using the Shapiro–Wilk test. For comparisons between two groups, the unpaired Student’s *t*-test was used for normally distributed data; otherwise, the non-parametric Mann–Whitney U test was applied. For comparisons among multiple groups, one-way analysis of variance (ANOVA) followed by Tukey’s post hoc test was used for data meeting parametric assumptions; otherwise, the non-parametric Kruskal–Wallis H test followed by Dunn’s post hoc test was employed. Categorical data were analyzed using the chi-square test. A *p*-value of <0.05 was considered statistically significant. Graphs were generated using GraphPad Prism software (version 8.0).

## 5. Conclusions

This study provides a theoretical basis for the overall efficacy of TFSC in the treatment of vascular dementia. Firstly, the composite model of hypertension and chronic hypoperfusion used in this study allows the therapeutic effect of the drug to be investigated along the dimension of disease comorbidity, consistent with the experimental purpose of exploring its overall efficacy. Secondly, it systematically demonstrates, across multiple dimensions (including hemodynamics, behavior, histopathology, serum biochemistry, and transcriptomics), that the natural extract TFSC has the potential to regulate vascular function and simultaneously protect the nervous system. These findings offer new experimental clues for exploring treatment strategies that integrate the management of vascular risk factors with direct neural intervention for VaD.

## Data Availability

The original contributions presented in this study are included in the article/Supplementary Materials. Further inquiries can be directed to the corresponding authors.

## Supplementary Materials

The following supporting information can be downloaded at https://www.mdpi.com/article/10.3390/ph19050700/s1: Table S1: Sequences of the real-time PCR primers used in this study; Table S2: Qualitative analysis of components in TFSC by UPLC-Q-TOF-MS/MS; Figure S1: UHPLC-DAD chromatogram of the mixed reference standard solution (top) and the TFSC (bottom); Figure S2: Body weight measurements of animals during the experimental period; Figure S3: Bar chart of the number of upregulated and downregulated DEGs in the TFSC groups compared with the Model group (PDF).

## Abbreviations

AD: Alzheimer’s disease; Ang-II, angiotensin II; BP, Blood pressure; CAT, Catalase; CBF, Cerebral blood flow; CC, corpus callosum; DEGs, Differentially expressed genes; DG, dentate gyrus; GO, Gene Ontology; GSH, Glutathione; H&E, Hematoxylin-Eosin; IL-1β, Interleukin-1β; IL-6, Interleukin-6; KEGG, Kyoto Encyclopedia of Genes and Genomes; MWM, Morris water maze; NBP, N-butylphthalide; NOR, Novel object recognition; OFT, Open field test; RI, recognition index; ROS, Reactive Oxygen Species; SC, Snow Chrysanthemum; SHR, Spontaneously hypertensive rats; SOD, Superoxide dismutase; TFSC, Total flavonoids of Snow Chrysanthemum; TNF-α, Tumor necrosis factor-α; VaD, Vascular dementia.
