# Supplementary material for: Total Flavonoids from Snow Chrysanthemum Exert Synergistic Vascular and Neuroprotective Effects in Hypertensive Vascular Dementia Rats
Source: Pharmaceuticals (Basel). 2026 Apr 29;19(5):700. doi: 10.3390/ph19050700 (PMC13210286; doi:10.3390/ph19050700)
Supplement: Supplementary file 1 [file pharmaceuticals-19-00700-s001.zip › pharmaceuticals-4265655-supplementary.pdf]

Supplementary Table S1. Sequences of the real-time PCR primers used in this study.

Gene	Forward primer	Reverse primer
Tnfrsf25	5′-TGTGGCTGGAAGCAGATGTT-3′	5′-GGGTCTCCATCCCAGCTATG-3′
Crygd	5′-GCCATGGGGAAGATCACCT-3′	5′-CGACGAAGGAAGTACTGGCA-3′
Sox15	5′-GGTCCATTTCAGCCAGGGAG-3′	5′-GTCTGCAATGGGAAGAGGTGTA-3′
Cd180	5′-TCCTGGCACTCTACCAAACT-3′	5′-ATCCAGTAAATCTGGCACCTGG-3′
Tcf19	5′-AAGTCTGGCTGCACCTATCG-3′	5′-TGACCAAAGTCCCTTGGCTG-3′
Nkd1	5′-TGGAGAAACTGAGCGAACCG-3′	5′-AGCTGGTAATGTCCTCACGG-3′
Klf4	5′-GTGCCCCGACTAACCGTTG-3′	5′-CCGATTCCTGGTGGGATAGC-3′
P2ry2	5′-CGGGGACCTAAAGAGGAACG-3′	5′-CACGTAATGGGCTCTCCCTG-3′
IL-6R	5′-TGGACTACCACGGGAAACA-3′	5′-GGATGCCACTCACAAAAGG-3′
Hspb1	5′-TGGAGATCACTGGCAAGCAC-3′	5′-GCCTCGAAAGTGACCGGAAT-3′
ACTB	5′-CGTTGACATCCGTAAAGACC-3′	5′-CTAGGAGCCAGGGCAGTAA-3′

Supplementary Table S2. Qualitative analysis of components in TFSC by UPLC Q TOF MS/MS.

NO.	Rt (min)	Compounds	Formula	Class	molecular weight	$[M-H]^-$ (*m/z*)		$[M+H]^+$ (*m/z*)		Error(-) (ppm)
						Measured	MS/MS fragments/adduct ion	Measured	MS/MS fragments/adduct ion	
1	1.39	unknow				266.0936	312.1009	268.1467	136.1079	
2	3.35	linocinnamarin	$C_{16}H_{20}O_8$	phenylpropanoid	340.1158	339.0798	177.0208			-10.9
3	3.49	protocatechuic acid	$C_7H_6O_4$	phenolic acid	154.0266	153.0202				9.0
4	3.63	unknow				527.1112	465.1104, 365.1474, 347.0512		367.1884	
5	3.82	(2R,3R)-dihydroquercetin-7-O-β-D-glucopyranoside or its analogues	$C_{21}H_{22}O_{12}$	flavone	466.1111	465.1104	447.1073, 303.0558	467.1440	305.1090	-3.4
6	3.94	unknow				203.0841	333.0480	205.1449	188.1167	
7	4.14	okanin-4'-O-β-D-glucopyranoside/2R/S-3',4',8-trihydroxyflavanone-7-O-glucoside/2R/S-eriodictyol-7-glucoside/5,7,3′,5′-tetrahydroxyflavanone-7-O-β-D-glucopyranoside or its analogues	$C_{21}H_{22}O_{11}$	flavone	450.1162	449.1154	393.1823, 373.1250, 287.0583			3.8
8	4.78	4-hydroxybenzoic acid	$C_7H_6O_3$	phenolic acid	138.0316	137.0263				18.0
9	5.09	(2R,3R)-dihydroquercetin-7-O-β-D-glucopyranoside or its analogues	$C_{21}H_{22}O_{12}$	flavone	466.1111	465.1104	931.2252, 303.0558	467.1484	305.1090, 933.1990, 603.1065	-3.4
10	5.44	chlorogenic acid/cryptochlorogenic acid/neochlorogenic acid	$C_{16}H_{18}O_9$	phenylpropanoid	354.0951	353.0924	707.1957, 191.0594	355.1399	163.0851	-10.2
11	6.50	(2R,3R)-dihydroquercetin-7-O-β-D-glucopyranoside or its analogues	$C_{21}H_{22}O_{12}$	flavone	466.1111	465.1104	303.0558, 285.0429	467.1484	305.1090	-3.4
12	8.31	sikokianin D/sikokianin E	$C_{42}H_{42}O_{22}$	flavone	898.2168	897.2210		899.1971	737.1684,469.1182	3.8

NO.	Rt (min)	Compounds	Formula	Class	molecular weight	[M−H]⁻ (*m/z*) Measured	MS/MS fragments/adduct ion	[M+H]⁺ (*m/z*) Measured	MS/MS fragments/adduct ion	Error(-) (ppm)
13	8.95	okanin-4'-O-β-D-glucopyranoside/2R/S-3',4',8-trihydroxyflavanone-7-O-glucoside/2R/S-eriodictyol-7-glucoside/5,7,3′,5′-tetrahydroxyflavanone-7-O-β-D-glucopyranoside/ or its analogues	$C_{21}H_{22}O_{11}$	flavone	450.1162	449.1154	899.2336	451.1524	289.1145	3.8
14	9.27	okanin-4'-O-β-D-glucopyranoside/2R/S-3',4',8-trihydroxyflavanone-7-O-glucoside/2R/S-eriodictyol-7-glucoside/5,7,3′,5′-tetrahydroxyflavanone-7-O-β-D-glucopyranoside/ or its analogues	$C_{21}H_{22}O_{11}$	flavone	450.1162		311.0817	451.1568	289.1145, 739.1962	3.8
15	9.35	unknow				448.1093	897.2210, 591.2761			
16*	9.91	flavanomarein (isookanin-7-O-β-D-glucoside)	$C_{21}H_{22}O_{11}$	flavone	450.1162	449.1154	899.2336, 1349.3683	451.1568	289.1145, 739.1862, 901.2166	-14.5
17	10.37	sikokianin D/sikokianin E	$C_{42}H_{42}O_{22}$	flavone	898.2168	897.2210	448.1093, 581.1633, 377.1899	899.2032	737.1684, 469.1182, 575.1417	-1.6
18*	10.95	isocoreopsin (Butein-4-O-β-D-glucopyranoside)	$C_{21}H_{22}O_{10}$	flavone	434.1213	433.1233	611.1761, 867.2465	435.1619	273.1211, 613.1868, 869.2308	-8.5
19	11.28	okanin-4'-O-β-D-glucopyranoside/2R/S-3',4',8-trihydroxyflavanone-7-O-glucoside/2R/S-eriodictyol-7-glucoside/5,7,3′,5′-tetrahydroxyflavanone-7-O-β-D-glucopyranoside	$C_{21}H_{22}O_{11}$	flavone	450.1162	449.1154	433.2213			3.8
20	11.56	isoquercitrin	$C_{21}H_{20}O_{12}$	flavone	464.0955	463.0946	507.1655, 439.191	465.1342	547.1370, 303.0933	-14.3

NO.	Rt (min)	Compounds	Formula	Class	molecular weight	[M−H]⁻ (*m/z*)		[M+H]⁺ (*m/z*)		Error(-) (ppm)
						Measured	MS/MS fragments/adduct ion	Measured	MS/MS fragments/adduct ion	
21	11.62	hyperoside	$C_{21}H_{20}O_{12}$	flavone	464.0955	463.0990				-4.8
22	11.90	unknow				611.1710	395.1986	613.1868	265.2100	
23*	12.90	quercetagetin-7-O-β-D-glucoside	$C_{21}H_{20}O_{13}$	flavone	480.0904	479.0923	959.1858, 525.0966, 317.0360	481.1272	319.0882, 961.1543	2.1
24	13.45	4-[(2E)-3-(3,4- dihydroxyphenyl)-2-propenoyl]-3-hydroxyphenyl β-D-glucopyranoside	$C_{21}H_{22}O_{10}$	flavone	434.1213	433.1233				-8.5
25*	13.91	isookanin	$C_{15}H_{12}O_6$	flavone	288.0634	287.0618	575.1325	289.1145		-8.7
26	14.61	unknow				611.1710		613.1868		
27	15.24	taxifolin/8-hydroxyfustin/3,5,7,3',5'-pentahydroxydihydroflavone/3,5,6,7,4'-pentahydroxydihydroflavone	$C_{15}H_{12}O_7$	flavone	304.0583	303.0558	607.1234, 285.0464	305.1090		17.0
28	15.83	okanin-4'-O-β-D-glucopyranoside/2R/S-3',4',8-trihydroxyflavanone-7-O-glucoside/2R/S-eriodictyol-7-glucoside/5,7,3′,5′-tetrahydroxyflavanone-7-O-β-D-glucopyranoside	$C_{21}H_{22}O_{11}$	flavone	450.1162	449.1198	403.1674	451.1568		-4.7
29	16.15	okanin-4'-O-β-D-glucopyranoside/2R/S-3',4',8-trihydroxyflavanone-7-O-glucoside/2R/S-eriodictyol-7-glucoside/5,7,3′,5′-tetrahydroxyflavanone-7-O-β-D-glucopyranoside	$C_{21}H_{22}O_{11}$	flavone	450.1162	449.1198	899.2336, 287.0618	451.1568	289.1145, 901.2104	-4.7

NO.	Rt (min)	Compounds	Formula	Class	molecular weight	$[M-H]^-$ (*m/z*)		$[M+H]^+$ (*m/z*)		Error(-) (ppm)
						**Measured**	**MS/MS fragments/adduct ion**	**Measured**	**MS/MS fragments/adduct ion**	
30	16.51	taxifolin/8-hydroxyfustin/3,5,7,3',5'-pentahydroxydihydroflavone/3,5,6, 7,4'-pentahydroxydihydroflavone	$C_{15}H_{12}O_7$	flavone	304.0583	303.0558				17.0
31*	16.72	quercetagitrin	$C_{21}H_{20}O_{12}$	flavone	464.0955	463.0990	927.1995, 509.1039	465.1342	303.0933, 929.1661	-4.8
32*	17.22	kaempferol 7-O-glucoside	$C_{21}H_{20}O_{11}$	flavone	448.1006	447.1043	895.2044, 285.0464	449.1384	287.1004, 465.1342, 897.1799	6.5
33*	17.75	marein	$C_{21}H_{22}O_{11}$	flavone	450.1162	449.1198	899.2398, 1349.3683	451.1568	289.1145	-4.7
34	18.96	unknow				493.1131	539.1188	495.1391	333.1041	
35	19.75	unknow					610.4272, 495.1566, 449.1154	283.7615	566.4463, 588.4255	
36	20.23	okanin-4'-O-β-D-glucopyranoside/2R/S-3',4',8-trihydroxyflavanone-7-O-glucoside/2R/S-eriodictyol-7-glucoside/5,7,3′,5′-tetrahydroxyflavanone-7-O-β-D-glucopyranoside	$C_{21}H_{22}O_{11}$	flavone	450.1162	449.1198		451.1524	597.1924, 289.1145	-4.7
37	20.65	3-O-caffeoyl quinic acid butyl ester	$C_{20}H_{26}O_9$	flavone	410.1577		491.1282, 421.2491, 193.0541	411.2378	249.1938, 234.1302, 451.2220	
38	20.95	coretinphenol	$C_{21}H_{32}O_9$	terpenoid	428.2046	427.2079	265.1492			-5.6
39	21.18	dicaffeoylquinic acid	$C_{25}H_{24}O_{12}$	phenylpropanoid	516.1268	515.1320	321.1616			-1.0
40	21.48	(2R,3R)-3,5,7,4'-tetrahydroxydihydroflavone/(2S)-5,7,3',5'-tetrahydroxydihydroflavone	$C_{15}H_{12}O_6$	flavone	288.0634	287.0618				-25.4
41	21.71	2S-7,3',5'-trihydroxydihydroflavone/naringetol/naringenin	$C_{15}H_{12}O_5$	flavone	272.0685	271.0664	491.1282, 377.1899	273.1211		-28.4
42	22.09	dicaffeoylquinic acid	$C_{25}H_{24}O_{12}$	phenylpropanoid	516.1268	515.1320	1031.2651, 353.0963	517.1572	499.1487, 1033.2156, 298.6008	-1.0

NO.	Rt (min)	Compounds	Formula	Class	molecular weight	[M−H]$^-$ (*m/z*) Measured	[M−H]$^-$ (*m/z*) MS/MS fragments/adduct ion	[M+H]$^+$ (*m/z*) Measured	[M+H]$^+$ (*m/z*) MS/MS fragments/adduct ion	Error(-) (ppm)
43	22.39	unknow				527.2241	573.2338, 1101.4529, 1055.4438		349.2043, 284.1373, 511.2423, 548.2191, 1057.3978	
44*	23.02	7,8,3',4'-tetrahydroxyflavanone	$C_{15}H_{10}O_6$	flavone	286.0477	285.0464	641.1304, 371.1422, 571.0984	287.0970	643.14	-24.6
45	23.68	coretincone /prunin/flavanocorepsin	$C_{21}H_{22}O_{10}$	flavone	434.1213	433.1233		435.1448	273.1211	-8.5
46	23.90	coretincone /prunin/flavanocorepsin	$C_{21}H_{22}O_{10}$	flavone	434.1213	433.1233	867.2526	435.1619	273.1211	-8.5
47	24.58	unknow					723.5199		340.3005, 679.5178, 359.2733, 701.4996	
48	24.97	dicaffeoylquinic acid	$C_{25}H_{24}O_{12}$	phenylpropanoid	516.1268	515.1320		517.1572	499.1487, 298.6008, 163.0851, 1033.2156, 794.1595	-1.0
49*	25.68	okanin	$C_{15}H_{12}O_6$	flavone	288.0634	287.0618		289.1145		-25.4
50	26.68	unknow				471.1973	517.2020		967.1766	
51	27.37	coreoside D	$C_{20}H_{28}O_8$	polyacetylene	396.1784		836.6025	396.8370	792.5863, 814.5659, 415.8099	
52	27.49	unknow				471.1973	517.2020		493.1608, 289.1145	
53	28.47	6′-acetyl marein	$C_{23}H_{24}O_{12}$	flavone	492.1268	491.1282				-8.8
54	28.84	eriodictyol	$C_{15}H_{12}O_6$	flavone	288.0634	287.0618	575.1276, 419.1422	289.1145		-25.4
55	29.90	dihydromarein	$C_{21}H_{24}O_{11}$	flavone	452.1319		949.6851, 359.1780, 201.1164	453.3757	905.6564	
56	31.83	maritimetin/luteolin/kaempferol/Lunatin/Omega-Hydroxyemodin	$C_{15}H_{10}O_6$	flavone	286.0477	285.0464		287.1004	421.6836	-24.6
57	32.30	unknow			508		1062.7668	509.9109	340.3005	
58*	32.66	butin	$C_{15}H_{12}O_5$	flavone	272.0685	271.0664				-10.7
59	34.20	3-O-caffeoyl quinic acid butyl ester	$C_{20}H_{26}O_9$	polyacetylene	410.1577	409.1588				-11.2
60	34.38	unknow				582.2744		584.2939	438.2701, 311.6584	
61	34.52	unknow					598.2717		600.2861, 454.2648, 340.1646	

NO.	Rt (min)	Compounds	Formula	Class	molecular weight	[M−H]⁻ (*m/z*)		[M+H]⁺ (*m/z*)		Error(-) (ppm)
						**Measured**	**MS/MS fragments/adduct ion**	**Measured**	**MS/MS fragments/adduct ion**	
62	34.63	2S-7,3',5'-trihydroxydihydroflavone/naringetol/naringenin	$C_{15}H_{12}O_5$	flavone	272.0685	271.0664				-28.4
63*	34.84	butein	$C_{15}H_{12}O_5$	flavone	272.0685	271.0698	582.2744	273.1211	584.2939	-15.9
64	35.15	unknow				973.5576		975.5167		
65	35.24	sulphuretin/7,3',4'-trihydroxyflavone/apigenin/Emodin	$C_{15}H_{10}O_5$	flavone	270.0528	269.0509	425.1917, 959.4458	271.1063	961.4080, 500.2280, 403.2079	-28.2
66	35.39	unknow				582.2793	628.2814, 1165.5485, 462.2138	584.2939	438.2701	
67	35.52	unknow				885.5027		887.4750	463.2653	
68	35.60	unknow				612.2852	885.5027, 553.1476	614.3018	887.4750, 555.1675	
69	35.67	unknow				511.2312	557.2386，1069.4723，1023.4686		351.2182, 1025.4113, 532.2280, 276.1385, 788.3069	
70	35.92	unknow				801.3719	329.2439, 591.2263	803.3529	402.2226, 657.3350	
71	35.99	unknow				797.4528	785.3743, 553.2054, 327.2260	799.4378	787.3594, 419.2337	
72	36.15	unknow				785.3743	425.1959, 327.2260	787.3594	641.3409, 394.2207	
73	36.46	unknow				785.3743	831.3815	787.3651	394.2248, 641.3461,	
74	36.58	unknow				815.3895	861.3958	817.3651	409.2281, 671.3508, 961.4080	
75	37.48	unknow						762.4160		
76	37.55	unknow				553.2439	599.2537, 431.2462			
77	39.55	unknow					387.2973	343.3340	240.2775	

Note: Compounds marked with an asterisk (*) have been verified through reference standard comparison.

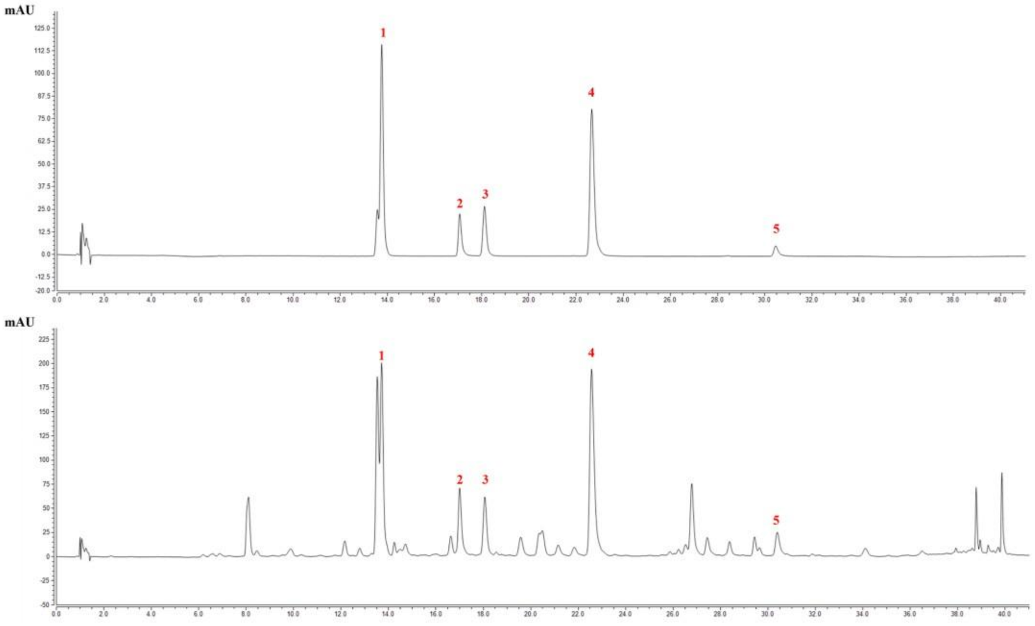

Supplementary Figure S1 . UHPLC-DAD chromatogram of the mixed reference standard solution (top) and the TFSC (bottom). The identified peaks, confirmed by comparison with reference standards, correspond to isookanin-7-O- β -D-glucoside (1), quercetagetin-7-O-β-D-glucoside (2), isookanin (3), marein (4), and okanin (5).

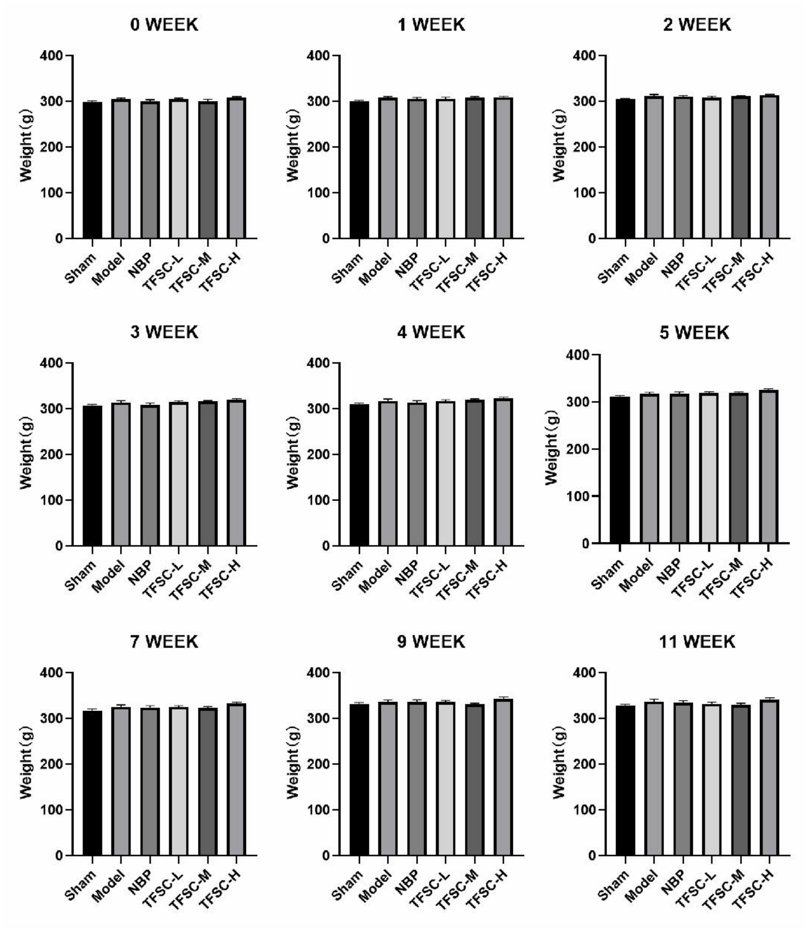

Supplementary Figure S2. Body weight measurements of animals during the experimental period

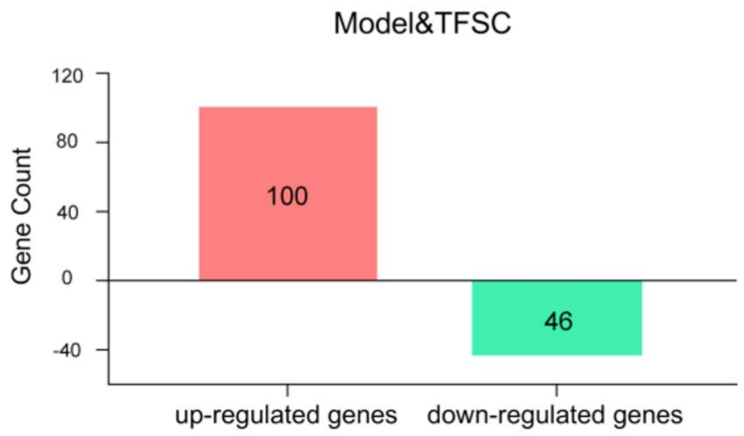

Supplementary Figure S3. Bar chart of the number of upregulated- and downregulated DEGs in the TFSC groups compared with Model group
